# Propionate attenuates osteoarthritis progression by regulating the gut-joint axis

**DOI:** 10.3389/fimmu.2026.1717556

**Published:** 2026-03-11

**Authors:** Segyeong Han, Keun-Hyung Cho, Hyun Sik Na, JooYeon Jhun, Young-Mee Moon, Jeong Won Choi, Seok Jung Kim, Mi-La Cho

**Affiliations:** 1 Lab of Translational ImmunoMedicine (LaTIM), Catholic Research Institute of Medical Science, College of Medicine, Catholic University of Korea, Seoul, Republic of Korea; 2 Department of Medical Sciences, Graduate School of Catholic University of Korea, Seoul, Republic of Korea; 3 Department of Pathology, College of Medicine, Catholic University of Korea, Seoul, Republic of Korea; 4 Department of Orthopedic Surgery, Uijeongbu St. Mary’s Hospital, College of Medicine, Catholic University of Korea, Seoul, Republic of Korea

**Keywords:** autophagy, inflammation, MIA, osteoarthritis, propionate

## Abstract

**Introduction:**

Osteoarthritis (OA) is a degenerative joint disorder characterized by cartilage degradation, inflammation, and pain. Growing evidence indicates that dysregulation of the gut–joint axis contributes to OA progression. This study investigated the therapeutic potential of propionate, a gut-derived short-chain fatty acid, in OA.

**Methods:**

A monosodium iodoacetate (MIA)-induced OA rat model was used to evaluate the effects of propionate on pain and inflammation through behavioral assessments, histological analysis, and gut microbiota profiling. The intestinal environment was further assessed by histology, tight junction protein analysis, and microbiota characterization. Human OA chondrocytes were analyzed using qPCR and RNA sequencing following IL-1β stimulation with or without propionate treatment.

**Results:**

Propionate attenuated OA severity in MIA-induced rats by improving pain behaviors, preserving cartilage structure, reducing nociceptive and inflammatory markers, and restoring intestinal barrier function and microbial balance. In human OA chondrocytes, propionate modulated inflammatory and ECM-related gene expression, promoted autophagy, and suppressed catabolic and inflammatory cell death pathways, highlighting its therapeutic potential in OA.

**Discussion:**

Propionate, a gut-derived SCFA, alleviated pain, protected cartilage, reduced inflammation, restored gut barrier integrity, and rebalanced microbiota in OA rats. In human OA chondrocytes, it upregulated ECM-related genes, downregulated inflammatory mediators, and enhanced autophagy. These findings suggest that propionate may serve as a promising disease-modifying therapy for OA.

## 1 Introduction

Osteoarthritis (OA) is a chronic degenerative joint disease characterized by pain arising from cartilage degradation, osteophyte formation, subchondral bone sclerosis, microfractures, and synovial inflammation (1, 2). Although age and strenuous physical activity are established risk factors, OA pathogenesis is multifactorial, involving mechanical, inflammatory, and metabolic processes. As the disease progresses, profound changes occur within the cartilage. Increasing evidence highlights the roles of altered immune responses, metabolic dysregulation, apoptosis, and impaired autophagy in chondrocytes and synovial cells as key contributors to OA development (3).

Recent evidence has highlighted the gut–joint axis as an important contributor to OA pathogenesis. Alterations in gut microbiota composition can promote systemic low-grade inflammation and metabolic imbalance, thereby exacerbating joint inflammation and cartilage damage (4–6). Gut-derived short-chain fatty acids (SCFAs) are central mediators of host–microbiota crosstalk, regulating intestinal barrier integrity and immune homeostasis. Dysregulated SCFA production may increase gut permeability and inflammatory signaling, allowing inflammatory cues to disseminate systemically and affect peripheral tissues, including articular cartilage (7–9). Emerging studies suggest that SCFAs modulate chondrocyte inflammation, extracellular matrix metabolism, and stress-adaptive pathways such as autophagy (10, 11).

The inflammatory response of chondrocytes represents a central pathological mechanism in OA. Rather than functioning solely as reactive cells, chondrocytes actively regulate disease progression by driving cartilage destruction through inflammatory and metabolic responses (12). Autophagy, a key process for maintaining cellular homeostasis, is frequently suppressed or impaired in OA chondrocytes, resulting in apoptosis and exacerbated inflammation. Activation of autophagy is essential for slowing OA progression, as it promotes chondrocyte survival, preserves extracellular matrix (ECM) integrity, and mitigates inflammation. For example, induction of autophagy through pathways such as mTOR inhibition or AMPK activation has been shown to attenuate OA development (13, 14).

In exploring the role of gut microbiota in OA, we identified microbial metabolites known as short-chain fatty acids (SCFAs) as key regulators of immune function, inflammation, and metabolism. Notably, we previously demonstrated that butyrate attenuates OA by enhancing autophagy in chondrocytes (15). However, emerging evidence suggests that propionate may offer distinct and broader therapeutic potential. While all SCFAs contribute to immune homeostasis, propionate has been shown to preferentially modulate systemic metabolic flux and neuro-inflammatory responses along the gut–brain–joint axis (16). Therefore, this study focused on propionate and its potential impact on osteoarthritis.

Recent studies have shown that dietary supplementation with probiotics can reshape the gut microbiota and suppress OA progression. Similarly, direct administration of SCFAs has been reported to restore immune homeostasis and reduce pain (15, 17). Clinical trials have further demonstrated that probiotic 3supplementation improved functional outcomes and pain control in patients with knee OA while attenuating chronic inflammation (18, 19). Together, these findings suggest that probiotics may confer therapeutic benefits by enriching SCFA-producing bacteria, with the resulting SCFAs contributing to the regulation of inflammation in OA.

Given that SCFAs such as propionate exert diverse modulatory effects on immune cells and cytokine production (20, 21), further investigation is warranted to clarify their roles in OA progression and their potential to regulate inflammatory responses in chondrocytes derived from OA patients. Building on this premise, the present study goes beyond simply confirming the effects of another microbial metabolite and instead aims to provide broader mechanistic insights into OA pathophysiology using human primary OA chondrocytes. Specifically, we examined how propionate regulates inflammatory signaling, ECM metabolism, and stress-adaptive pathways, including autophagy and inflammatory cell death, while integrating the analysis of pain-related neuro-inflammatory markers. In consideration of the clinical need for effective pain management in OA, celecoxib was included as a clinically relevant reference therapy widely used for OA-associated pain and inflammation. Furthermore, the mTOR signaling pathway, a central regulator of autophagy and chondrocyte homeostasis, was evaluated, and calcitonin gene-related peptide (CGRP) and transient receptor potential vanilloid 1 (TRPV1) were assessed as key mediators of nociceptive signaling and peripheral sensitization in OA. Accordingly, this study aims to determine whether propionate possesses pain-suppressing and inflammation-regulating functions and to elucidate its underlying mechanisms of action in human OA chondrocytes.

## 2 Materials and methods

### 2.1 Animals

Wistar rats (180–200 g) were obtained from Central Lab Animal Inc. (Seoul, South Korea). OA was induced by intra-articular injection of 50 µL of 1 mg monosodium iodoacetate (MIA; Sigma-Aldrich, St. Louis, MO, USA) into the right knee joint under 2% isoflurane anesthesia using a 26.5 G needle. Pain-related behaviors and weight-bearing (WB) were assessed three days after injection. Groups of 6 rats (n = 6 per group) were allocated based on baseline weight-bearing results. MIA-induced OA rats were orally administered propionate (80 mg/ml dissolved in Saline, Propionate; Sigma-Aldrich) or the positive control, celecoxib (50 mg/kg dissolved in 0.5% carboxymethylcellulose, CMC; Sigma-Aldrich), once daily for 23 days.

Pain responses were evaluated using mechanical allodynia and hind limb WB tests. Mechanical allodynia was assessed with a dynamic plantar aesthesiometer (Ugo Basile, Gemonio, Italy). Rats were acclimated on a metal mesh within acrylic chambers, and a 0.5-mm diameter monofilament probe was applied at a ramp speed of 25 s until paw withdrawal occurred. Paw withdrawal latency and paw withdrawal threshold were recorded automatically, with a maximum applied force of 50 g.

Hind limb weight distribution was assessed using an incapacitance meter (IITC Life Science, Woodland Hills, CA, USA). After a 5 min acclimation period in an acrylic restrainer, the ipsilateral and contralateral paws were positioned on dual force plates, and forces were recorded over 5 s. The mean value from three consecutive measurements was used to calculate the WB ratio.

All animal experiments were performed in accordance with the Animal Welfare Act and were approved by the Institutional Animal Care and Use Committee (IACUC) of the Catholic University of Korea, College of Medicine.

### 2.2 Histological analysis

#### 2.2.1 Tissue processing and histological scoring

Rat joint, intestine, and DRG tissues were collected on day 25 after MIA induction. Joint and intestine were paraffin-embedded and sectioned at 5 μm, and DRG was cryo-sectioned at 5 μm. Safranin O–stained joint sections were histologically evaluated for cartilage degeneration using both the Osteoarthritis Research Society International (OARSI) histopathology scoring system (grading 0-6) and the Mankin histological scoring system (total score range 0-14), which integrates assessment of structural integrity, proteoglycan depletion based on Safranin O staining, cellular abnormalities, and tidemark integrity for comprehensive cartilage evaluation (22, 23). H&E-stained intestine sections were quantified for barrier disruption via villus height/crypt ratios and inflammation grading. ZO-1 and occludin (OCLN) mean fluorescence intensity, measured via ImageJ thresholding on IF images, assessed tight junction integrity.

#### 2.2.2 Immunohistochemistry

Sections were treated with Proteinase K for antigen retrieval (6 min, RT), blocked with 3% H_2_O_2_, and incubated overnight at 4°C with primary antibodies diluted in Dako Antibody Diluent. HRP-labeled polymer secondary antibodies (EnVision+ System, Dako) were applied for 30 min, followed by DAB+ substrate for color development. Counterstaining was performed with Mayer’s hematoxylin. The antibodies used are listed in **Supplementary Table S1**. Antibody negative controls were included by omitting the primary antibody and using isotype-matched control IgG under identical staining conditions to verify antibody specificity and exclude non-specific background staining. Due to the structural complexity and large size of whole-joint sections, low-magnification IHC overview imaging was not performed, and representative regions were selected based on histological evaluation.

#### 2.2.3 Immunofluorescence

Intestinal tissues and chondrocytes were fixed and incubated with primary antibodies overnight at 4°C. Appropriate fluorescent secondary antibodies were applied for 2 h at room temperature, followed by nuclear staining with DAPI. Imaging was performed using ZEN 2012 software (Zeiss). The antibodies used are listed in **Supplementary Table S2**. Antibody negative controls were included by omitting the primary antibody and using isotype-matched control IgG under identical staining conditions to verify antibody specificity and exclude non-specific background staining. Stimulation and treatment conditions for confocal immunofluorescence experiments are specified in the corresponding figure legends.

### 2.3 Fecal DNA extraction, PCR amplification, and sequencing

Fecal samples were collected and stored at −70 °C. Fecal microbial DNA was extracted using the FastDNA^®^ SPIN Kit for Soil (MP Biomedicals) according to the manufacturer’s instructions. The V3–V4 regions of the bacterial 16S rRNA gene were amplified using fusion primers (341F and 805R) containing Illumina adapter sequences. PCR amplification was performed under standard cycling conditions, and amplicons were purified using magnetic bead-based cleanup followed by size selection to remove non-target fragments. Libraries were quantified and pooled prior to sequencing on an Illumina MiSeq platform (CJ Bioscience, Seoul, Korea). Raw sequencing reads were quality-filtered using Trimmomatic and merged using VSEARCH. Chimeric sequences were removed using the UCHIME algorithm, and operational taxonomic units (OTUs) were assigned based on the EzBioCloud 16S rRNA reference database. Alpha diversity indices and beta diversity distances (including Jensen–Shannon, Bray–Curtis, and UniFrac metrics) were calculated to evaluate microbial community structure. Differential taxonomic abundance was performed using LDA Effect Size (LEfSe) analysis with the Kruskal–Wallis test. All analyses were conducted using the EzBioCloud 16S-based MTP bioinformatics platform (CJ Bioscience, Seoul, Korea).

### 2.4 Primary chondrocyte isolation

Primary chondrocytes were isolated from the articular cartilage of 10 patients (3 males, 7 females who underwent total knee arthroplasty or joint replacement surgery at Seoul Uijeongbu St. Mary’s Hospital (IRB No. UC23TISI0069). The cartilage was sequentially digested with hyaluronidase, protease XIV, and collagenase V. Isolated chondrocytes were cultured in DMEM supplemented with 10% FBS in a 5% CO_2_ atmosphere until use in subsequent experiments. Passage 2–3 chondrocytes were used for all experiments.

### 2.5 qPCR analysis

Cells were stimulated with IL-1β (10 ng/mL), propionate (0.5 or 1 mM), or celecoxib (10 µM) for 24 h. RNA was extracted using TRIzol, reverse transcribed into cDNA with the Bio-Rad BN615 system and analyzed by qPCR using SYBR Green (Bio-Rad) with β-ACTIN as the internal control. Primer sequences are provided in **Supplementary Table S3**.

### 2.6 RNA sequencing

For RNA sequencing (Section 2.6), total RNA quality was assessed using NanoDrop and the Agilent 2100 Bioanalyzer. Libraries were prepared using the TruSeq Stranded mRNA LT Sample Prep Kit following the manufacturer’s protocol (Illumina, Part #15031047 Rev. E) and sequenced on an Illumina platform to generate paired-end reads (101 bp). Raw sequencing reads were subjected to quality assessment using FastQC (v0.11.7), followed by adapter and low-quality trimming using Trimmomatic (v0.38). Filtered reads were aligned to the reference genome (GRCh38_NCBI_109; annotation NCBI_109.20200522) using HISAT2 (v2.1.0) and Bowtie2 (v2.3.4.1). Transcript assembly and quantification were performed using StringTie (v2.1.3b), generating expression values including read counts, FPKM (Fragments Per Kilobase of transcript per Million mapped reads), and TPM (Transcripts Per Kilobase Million). Differentially expressed gene (DEG) analysis was conducted using edgeR, applying a threshold of |fold change| ≥ 2 and exactTest raw p-value < 0.05. Functional enrichment analysis was performed using KEGG pathway annotation. The sequencing dataset is available in the NCBI Sequence Read Archive under accession number PRJNA1336958.

### 2.7 Statistical analysis

Data was analyzed using GraphPad Prism 9.5. Pain and WB outcomes were assessed by two-way ANOVA followed by Bonferroni’s *post hoc* test. Group comparisons were performed using the Mann–Whitney or Kruskal–Wallis test, as appropriate. Data are presented as mean ± SEM or SD, and p < 0.05 was considered statistically significant.

## 3 Results

### 3.1 Propionate attenuates MIA-induced OA severity

**Figure 1:**
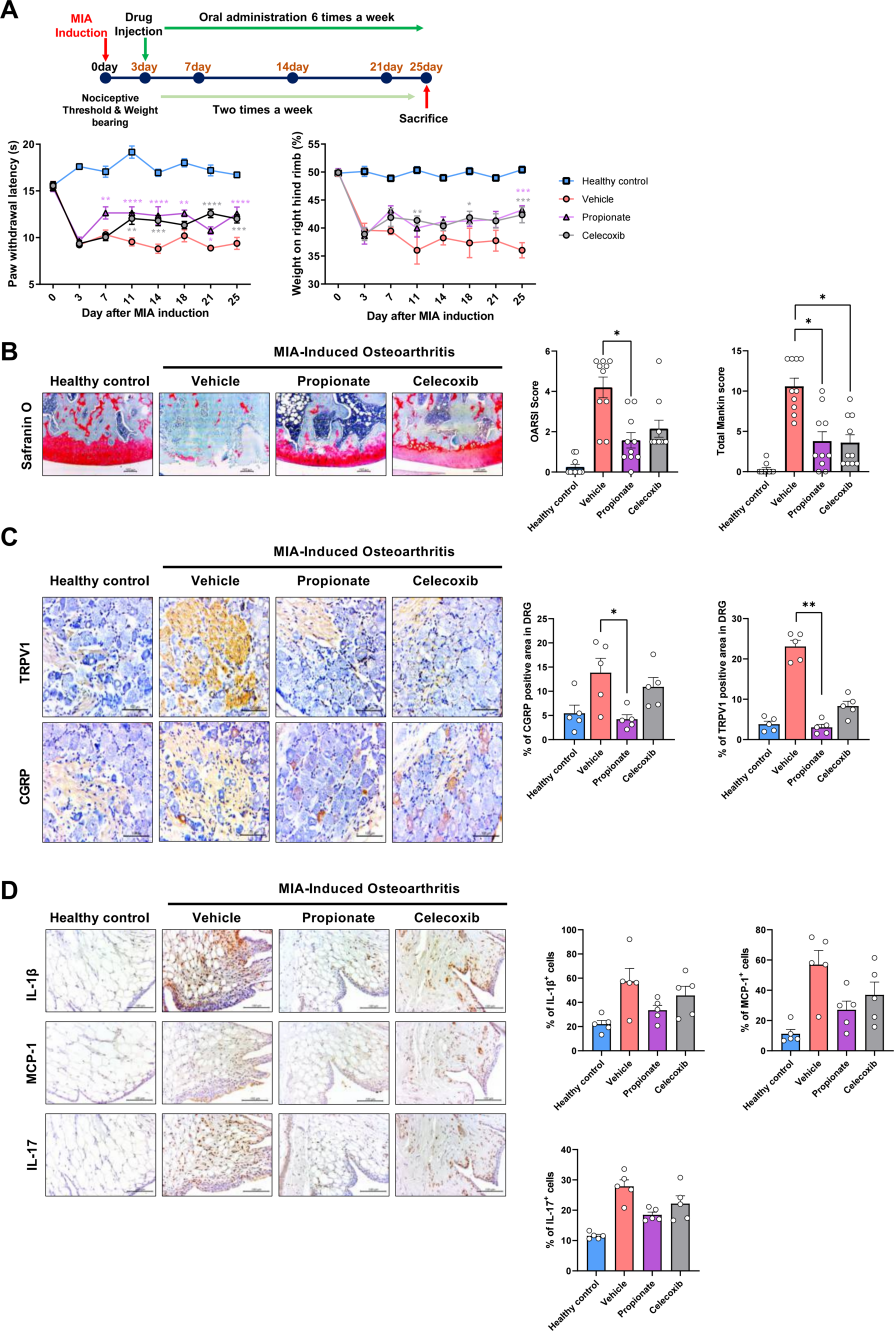
**(A)** Nociceptive behavior was assessed by measuring paw withdrawal latency (PWL) and weight-bearing (WB) over 25 days in Healthy Control, Vehicle, Propionate, and Celecoxib groups (n = 6 per group). **(B)** Cartilage integrity was evaluated by Safranin O staining of rat joints, followed by OARSI and Mankin scoring (magnification ×200). **(C)** TRPV1 and CGRP expression in rat DRG were detected by IHC (magnification 400×). **(D)** IL-1β, IL-17, and MCP-1 expression in rat synovium were detected by IHC (magnification 400×). Data are presented as mean ± SEM. Statistical significance was determined by two-way ANOVA with *post hoc* test **(A)** and Mann–Whitney test **(B–D)**. *p < 0.05, **p < 0.01, ***p < 0.001, ****p < 0.0001 vs. Vehicle.

To evaluate the therapeutic effects of propionate in MIA-induced OA, rats were intra-articularly injected with MIA and subsequently treated with propionate. OA progression was assessed through behavioral, histological, and molecular analyses. MIA injection significantly worsened these parameters, whereas propionate treatment restored them, compared with vehicle controls (**Figure 1A**). Cartilage integrity, examined by Safranin O staining and semi-quantitative scoring, revealed severe proteoglycan loss and cartilage degeneration in the MIA group, which were substantially alleviated by propionate. Both OARSI and Mankin scores were significantly lower in the propionate-treated group compared with vehicle (**Figure 1B**). Rat joint, intestine, and DRG tissues were collected on day 25 after MIA induction. To determine whether propionate modulates OA-related pain through neural pathways, nociceptive markers in the dorsal root ganglion (DRG) were analyzed by IHC. MIA injection increased TRPV1 and CGRP expression, indicating enhanced nociceptive signaling, whereas propionate treatment effectively suppressed their expression. (**Figure 1C**). In addition, analysis of joint tissues showed that propionate administration markedly reduced the expression of inflammatory mediators, including IL-1β, IL-17, and MCP-1, consistent with suppression of OA-associated inflammatory signaling within the joint microenvironment (**Figure 1D**).

### 3.2 Propionate improves intestinal integrity in MIA-induced OA

**Figure 2:**
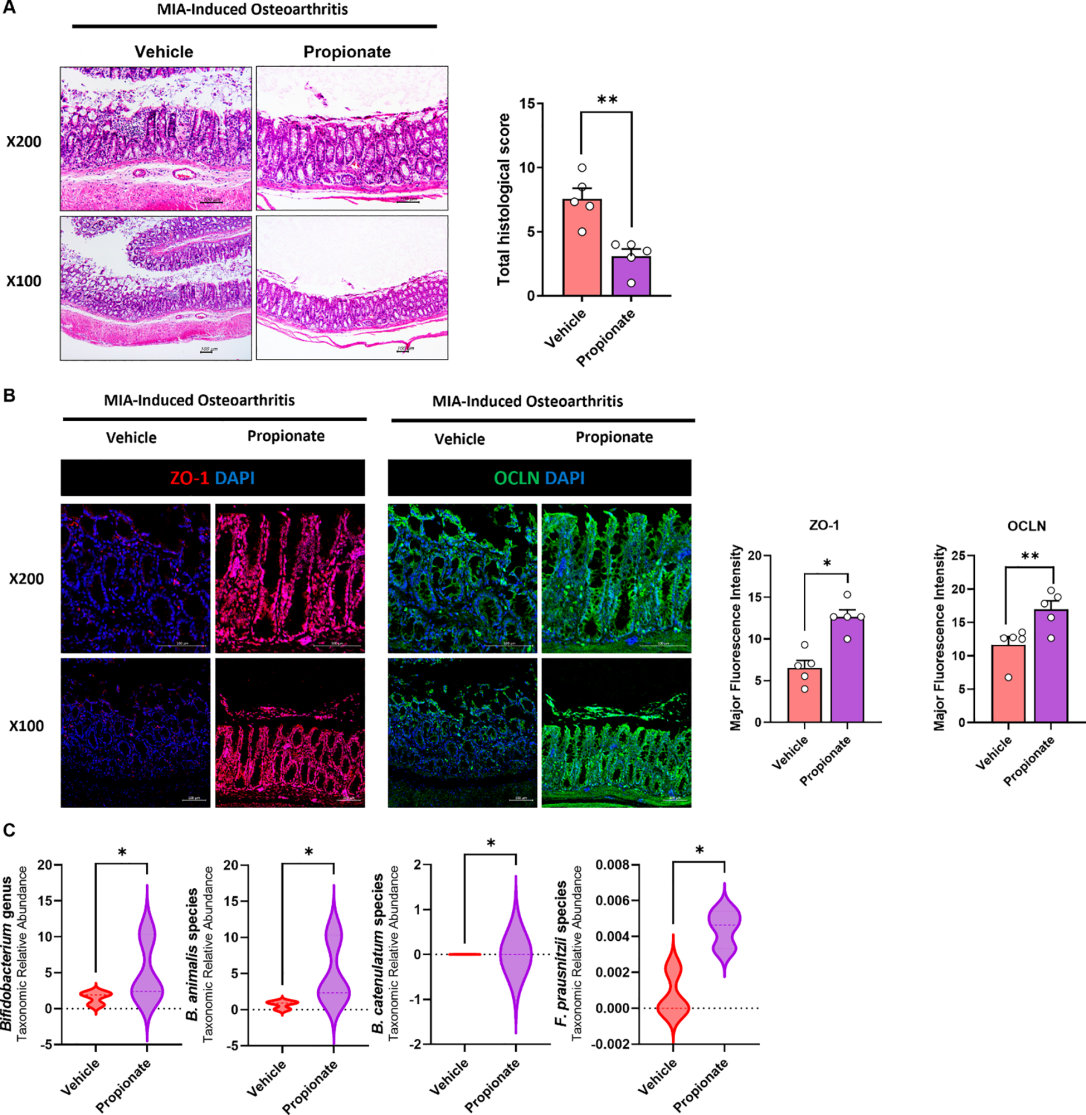
**(A)** Histological analysis of rat colon tissue was performed using H&E staining (magnification ×100, ×200). **(B)** Confocal microscopy of tight junction proteins ZO-1 (red), OCLN (green), and DAPI (blue) in rat colon tissues (magnification ×100, ×200). **(C)** Fecal microbiota composition in rats was determined by 16S rRNA sequencing, and differentially abundant taxa were identified by LEfSe analysis. Data are presented as mean ± SEM. Statistical significance was determined by the Mann–Whitney test. *p<0.05, **p<0.01 vs. Vehicle.

H&E staining revealed mucosal damage, epithelial disruption, and inflammatory cell infiltration in MIA-induced OA rats. These abnormalities were markedly reduced by propionate treatment, and the total histology score was significantly lower compared with vehicle controls (**Figure 2A**). Tight junction integrity, assessed by confocal microscopy for ZO-1 and OCLN, was markedly decreased in MIA-induced rats, indicating impaired intestinal barrier function. Propionate restored the expression of both proteins, suggesting improved epithelial integrity (**Figure 2B**). Gut microbiota profiling based on 16S rRNA sequencing revealed that MIA induction markedly altered microbial community structure, whereas propionate treatment partially restored microbial balance by enriching beneficial taxa associated with anti-inflammatory and metabolic homeostasis, including *Bifidobacterium animalis, Bifidobacterium catenulatum*, and *Faecalibacterium prausnitzii* (**Figure 2C**).

### 3.3 Propionate modulates ECM and inflammatory gene expression in human OA chondrocytes

**Figure 3:**
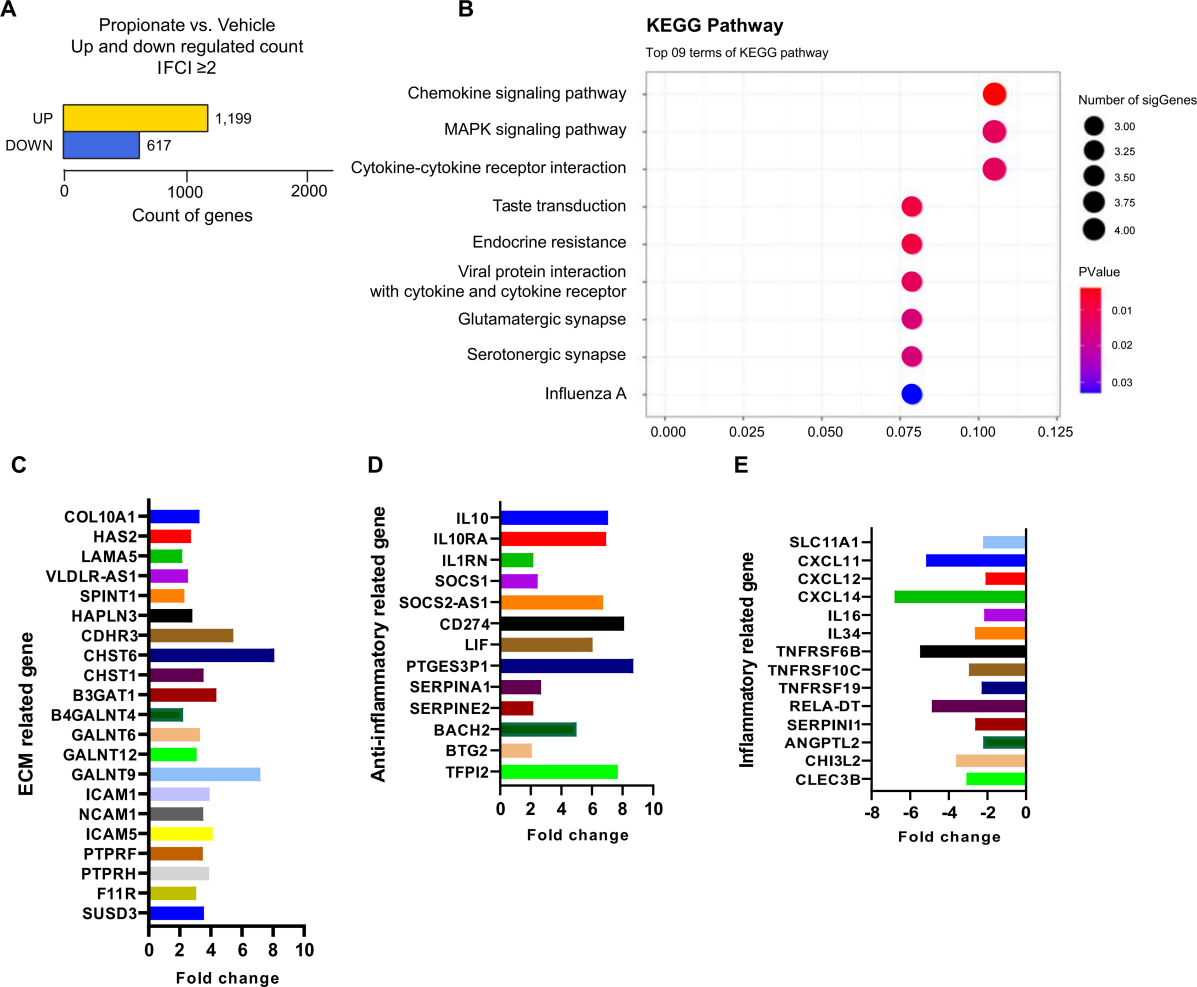
**(A)** Differential gene expression in human OA chondrocytes treated with or without propionate (|FC| ≥ 2). **(B)** Top nine enriched KEGG pathways. **(C)** ECM-related gene expression changes with propionate treatment. **(D)** Anti-inflammatory gene expression changes with propionate treatment. **(E)** Inflammatory gene expression changes with propionate treatment.

The direct effects of propionate on chondrocytes were evaluated by RNA sequencing following *in vitro* treatment. Propionate markedly altered gene expression compared with vehicle, with a significant increase in the number of upregulated genes (|FC| ≥ 2) (**Figure 3A**). KEGG pathway analysis revealed significant enrichment of OA-relevant pathways including chemokine signaling, MAPK signaling, and cytokine-cytokine receptor interactions (gene ratio: 0.1; **Figure 3B**), consistent with Propionate’s anti-inflammatory effects. Genes involved in ECM remodeling and adhesion, such as *SUSD3, PTPRH, ICAM* family members, *LAMA5*, and *COL10A1*, were upregulated after propionate treatment, suggesting a potential shift toward cartilage-protective remodeling (**Figure 3C**). Anti-inflammatory and immune-regulatory genes, including *SOCS1, SOCS2-AS1, IL1RN*, and *IL-10* signaling-related molecules, were also elevated (**Figure 3D**). Conversely, pro-inflammatory mediators such as *TNFRSF* family members and chemokines (*CXCL11, CXCL12*, and *CXCL14*) were significantly downregulated (**Figure 3E**).

### 3.4 Propionate restores autophagy activity in human OA chondrocytes and synovial tissue in OA animal model

**Figure 4:**
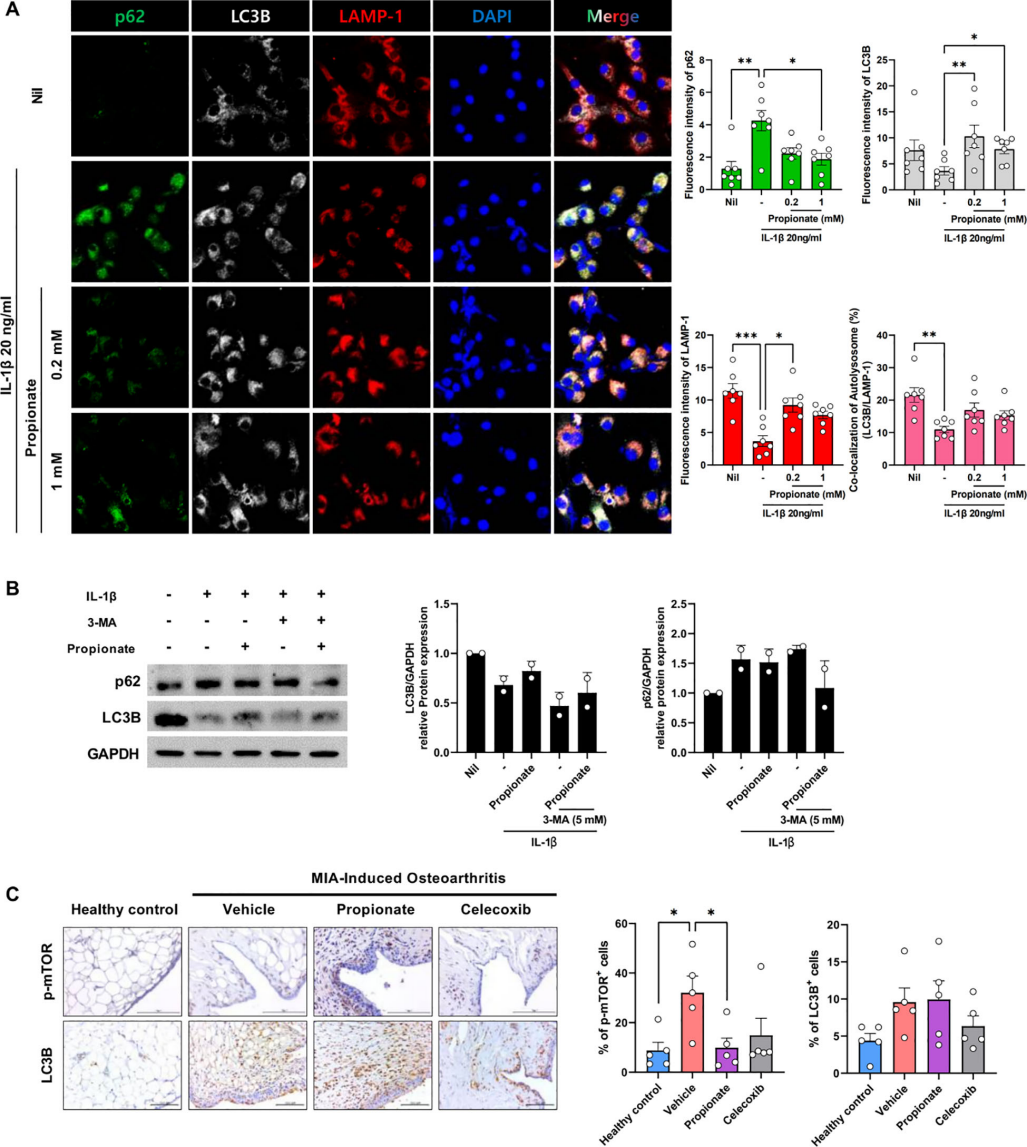
**(A)** Confocal microscopy of autophagy-related proteins p62 (green), LC3B (white), LAMP-1 (red), and DAPI (blue) in human OA chondrocytes stimulated with IL-1β (20 ng/mL) and treated with propionate (0.2 or 1 mM) (magnification ×400). Representative confocal images are shown (left), with corresponding quantitative analysis of fluorescence intensity (right). **(B)** OA chondrocytes were treated with IL-1β (20 ng/mL) in the presence or absence of propionate (0.2 mM) and the autophagy inhibitor 3-MA (5 mM). The expression of p62 and LC3B was analyzed by western blotting (left). GAPDH was used as a loading control. Qualification of LC3B/GAPDH and p62/GAPDH ratio is shown (right). **(C)** p-mTOR and LC3B expression in rat synovium were detected by IHC (magnification ×400). Data are presented as mean ± SD **(A)** and mean ± SEM **(B, C)**. Statistical significance was determined by Kruskal–Wallis or Mann–Whitney test. *p < 0.05, **p < 0.01, ***p < 0.001.

The mechanism of propionate action in chondrocytes was investigated by examining autophagy-related factors essential for chondrocyte survival under inflammatory conditions. Confocal microscopy revealed that propionate treatment decreased p62 and increased LAMP1 and LC3B expression in a dose-dependent manner, indicating enhanced autophagic flux. The LC3B/LAMP1 ratio was significantly higher in the propionate group compared with the vehicle, suggesting activation of autophagosome–lysosome fusion (**Figures 4A**, **Supplementary Figure S1**). To further assess whether autophagy activation contributes functionally to the protective effects of propionate, OA chondrocytes were stimulated with IL-1β in the presence or absence of the autophagy inhibitor 3-MA (5 mM). Western blot analysis showed that inhibition of autophagy by 3-MA reduced LC3B expression and induced accumulation of p62 under inflammatory conditions. Importantly, the propionate-induced restoration of autophagy markers was partially attenuated in the presence of 3-MA, supporting a functional link between autophagy activation and the protective effects of propionate (**Figure 4B**). *In vivo*, IHC analysis of joint tissues showed that the proportion of p-mTOR–positive cells within synovial tissue was markedly elevated in MIA-induced OA, whereas propionate treatment reduced p-mTOR expression to levels comparable with healthy controls. Conversely, LC3B-positive cells within synovial tissue increased following propionate treatment, indicating restoration of autophagic activity within the joint microenvironment (**Figure 4C**).

### 3.5 Propionate suppresses inflammatory mediators and inflammatory cell death in human OA chondrocytes and synovial tissue in OA animal model

**Figure 5:**
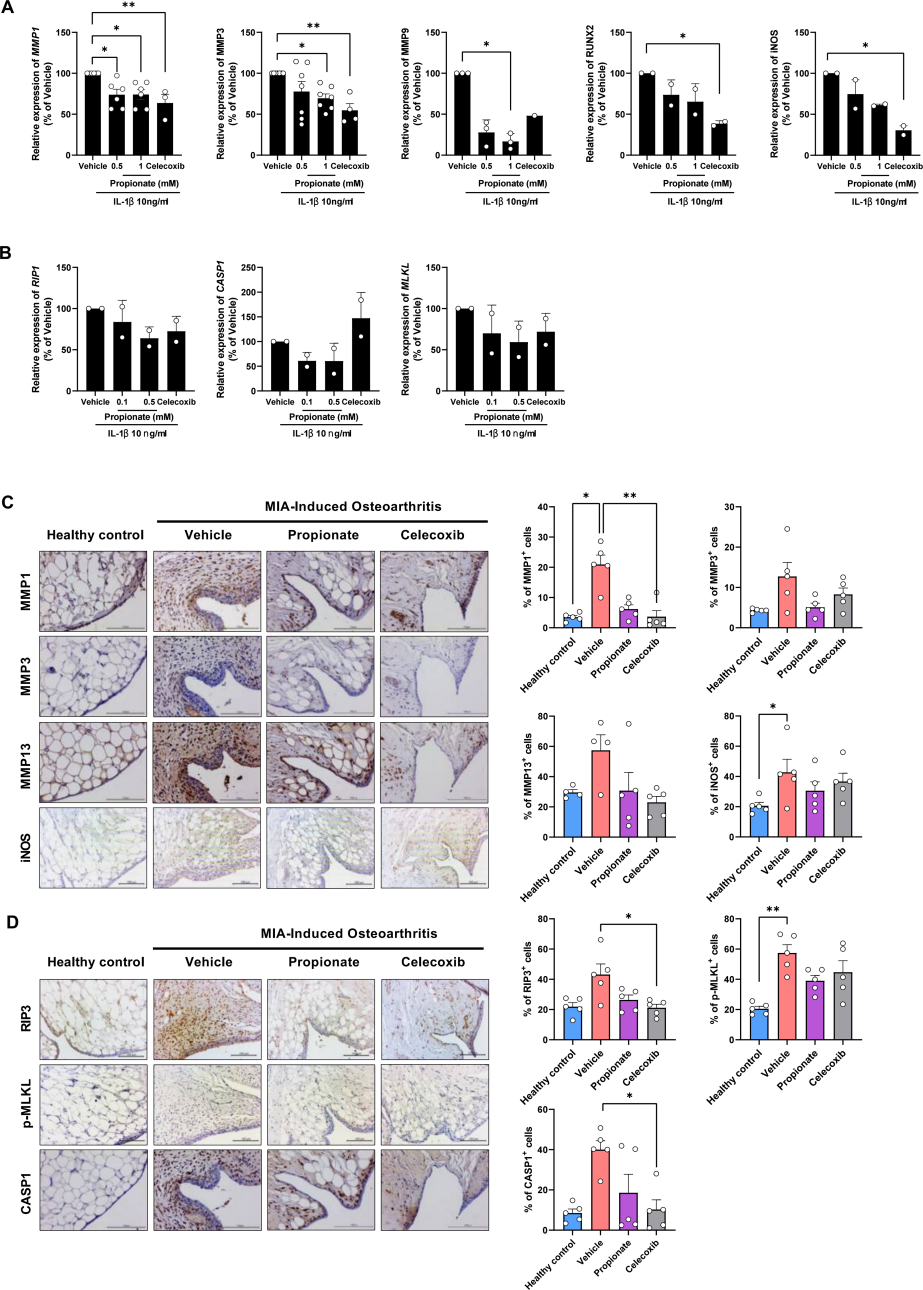
**(A)** qPCR analysis of inflammatory markers (*MMP1*, *MMP3*, *MMP9*, *RUNX2*, *iNOS*) in human OA chondrocytes treated with propionate or celecoxib. **(B)** qPCR analysis of inflammatory cell death–related markers (*RIP1*, *MLKL*, *CASP1*) in human OA chondrocytes treated with propionate or celecoxib. **(C)** Inflammatory markers (MMP1, MMP3, MMP13, iNOS) in rat synovium detected by IHC (magnification 400×). **(D)** Inflammatory cell death–related markers (RIP1, MLKL, CASP1) in rat synovium detected by IHC (magnification 400×). Data are presented as mean ± SD **(A, B)** and mean ± SEM **(C, D)**. Statistical significance was determined by the Mann–Whitney test. *p < 0.05, **p < 0.01.

The anti-inflammatory effects of propionate on chondrocytes were evaluated by assessing the expression and localization of inflammation-related markers. Propionate treatment significantly reduced mRNA levels of *MMP1, MMP3, MMP9, RUNX2*, and *iNOS*, whereas IHC confirmed decreased expression of MMP1, MMP3, MMP13, and iNOS (**Figures 5A, C**). These findings suggest that propionate mitigates catabolic and inflammatory responses in OA chondrocytes. Moreover, propionate treatment was associated with a downregulation of inflammatory cell death–related markers, as evidenced by the decreased mRNA expression of RIP1, MLKL, and CASP1. Consistent with these transcriptional changes, IHC analysis revealed a reduction in the staining intensity of RIP1, RIP3, MLKL, and mTOR (**Figures 5B, D**). These findings suggest that propionate contributes to the modulation of necroptotic and inflammatory pathways within the joint microenvironment To further validate suppression of inflammatory cell death at the signaling level, western blot analysis demonstrated that propionate significantly reduced phosphorylation of RIP1 and RIP3, key mediators of necroptosis activation (**Supplementary Figure S2**). These findings provide protein-level evidence supporting the inhibitory effect of propionate on inflammatory cell death signaling in OA chondrocytes.

## 4 Discussion

In our previous study, we showed that administration of inactivated Lactobacillus significantly altered the gut microbial profile in OA rats, notably increasing bacterial taxa involved in short-chain fatty acid production (15). Emerging evidence from gut–joint axis research highlights the pivotal role of the gut microbiota in joint health, suggesting that modulation of the intestinal environment may offer a novel therapeutic paradigm for OA beyond conventional pharmacological interventions (4, 24). To test whether propionate, a major SCFA, exerts therapeutic effects on OA, we administered propionate to an MIA-induced OA model. Propionate supplementation alleviated pain and improved weight-bearing, whereas *in vitro* treatment of human OA chondrocytes enhanced autophagy, suppressed pro-inflammatory mediators, and modulated inflammatory cell death. Together, these findings indicate that propionate exerts both systemic and chondrocyte-intrinsic effects relevant to OA pathophysiology.

SCFAs, the fermentation products of gut microbiota, have been widely studied for their anti-inflammatory and immunomodulatory properties across various diseases, including autoimmune disorders and metabolic syndromes (20). SCFAs modulate inflammatory responses in immune cells, promote the production of anti-inflammatory cytokines such as IL-10 (24–27), and suppress autoimmune pathology in experimental models of EAE, colitis, IBD, and CIA (28–31). In our previous work, we demonstrated that butyrate attenuated OA progression by suppressing NF-κB and MAPK signaling, thereby alleviating inflammatory responses, inhibiting matrix degradation, and restoring autophagic activity in chondrocytes. Notably, those previous observations were largely confined to chondrocyte-intrinsic mechanisms regulating cartilage homeostasis. In contrast, while the role of propionate—another major SCFA in osteoarthritis (OA) has remained less explored, our present findings indicate that its impact extends far beyond cartilage-centered pathways. We demonstrate that propionate reinforces intestinal barrier integrity, as evidenced by the restored expression of ZO-1 and occludin, while coordinately modulating inflammatory signaling, extracellular matrix remodeling, and stress-adaptive pathways in human OA chondrocytes. Collectively, these results suggest that propionate functions not merely as a local chondroprotective agent, but as a systemic immunometabolic modulator that attenuates OA progression by strengthening the gut barrier and dampening inflammatory signaling upstream of joint pathology.

Using an MIA-induced OA rat model, we observed that propionate administration alleviated pain, preserved cartilage, and reduced inflammatory mediators. Because propionate can cross the blood–brain barrier and act on the peripheral nervous system, it may also influence the DRG. Indeed, we confirmed that CGRP and TRPV1, key pain signaling–related genes, were downregulated in the DRG of propionate-treated rats. Previous studies have shown that the SCFA receptor GPR41 in sympathetic ganglia regulates neural excitability and neurotransmitter release in response to SCFAs, particularly propionate (32). Our findings therefore suggest that propionate directly modulates neurological pain pathways to alleviate one of the major symptoms of OA: pain. In OA models, direct SCFA administration or conditions promoting SCFA production have been linked to an increased abundance of anti-inflammatory taxa such as *Bifidobacterium* and *Faecalibacterium* (33). Consistent with these reports, gut microbiota analysis in propionate-supplemented OA rats revealed enrichment of bacterial strains associated with the NF-κB signaling pathway, which regulates MMP-mediated cartilage degradation and inflammatory responses in OA. Together, these results suggest that propionate may attenuate OA progression through both neural mechanisms and microbiota-mediated immunometabolic pathways.

Propionate may also modulate OA progression through immune regulation. In the gut of OA animals treated with propionate, expression of the key tight junction proteins ZO-1 and OCLN was increased, suggesting enhanced intestinal barrier integrity and reduced systemic translocation of microbial products and inflammatory mediators. In addition, our study confirmed the anti-inflammatory effects of propionate on human OA chondrocytes. RNA sequencing revealed that propionate upregulated ECM–related genes critical for cartilage maintenance (*HAS2, HAPLN3, CHST1/6, B3GAT1*, and *GALNT6/9/12*), as well as anti-inflammatory mediators including *IL-*10, IL-10RA, IL-1Ra, SOCS1, *SERPINA1*, *SERPINE2*, and *TFPI2*. Conversely, expression of pro-inflammatory genes such as *CXCL11, CXCL12, CXCL14, IL-16, IL-34, TNFRSF6B*, and *SLC11A1* was markedly reduced. PCR analysis further confirmed downregulation of inflammatory factors, including *MMP1*, *MMP3*, *MMP9*, *RUNX2*, and *iNOS*. Collectively, these findings indicate that propionate exerts a dual protective effect in OA by promoting ECM homeostasis while suppressing inflammatory signaling in chondrocytes. In addition, the pathways with less direct relevance to osteoarthritis, such as taste transduction and Influenza A. Bitter taste receptors (TAS2Rs), however, are expressed beyond gustatory tissues and have been reported to regulate immune and inflammatory responses in various extra-oral cell types (34). In addition, enrichment of the “Influenza A” pathway often reflects activation of interferon-stimulated genes and stress-responsive transcriptional programs rather than virus-specific processes (35). Considering that short-chain fatty acids (SCFAs) can modulate IFN/STAT1-associated pathways in a context-dependent manner (36), these transcriptomic features may reflect broader cellular defense or stress-adaptive remodeling programs that intersect with the anti-inflammatory actions of SCFAs. Nevertheless, this interpretation remains exploratory and requires further targeted validation.

Propionate is known to enhance Treg activity and anti-inflammatory cytokine production, such as IL-10, via HDAC inhibition in T cells, while modulating antigen-presenting cells through GPR signaling (25, 37). In chondrocytes, butyrate has been reported to suppress inflammatory cytokine production through the GPR43 receptor (38). Furthermore, GPR41, a major SCFA receptor expressed in sympathetic and sensory ganglia, has been shown to regulate neuronal excitability and neurotransmitter release in response to propionate (32). Based on these observations, we propose a mechanistic hypothesis that propionate may exert its analgesic and anti-inflammatory effects through GPR41/43 signaling in the dorsal root ganglion and chondrocytes. Importantly, while our findings support a plausible neuro-inflammatory mechanism contributing to pain attenuation in OA, the present study did not directly characterize receptor-specific signaling using pharmacological antagonists or genetic knockout models. Therefore, the involvement of GPR41/43 should be regarded as a proposed model that warrants further experimental validation to definitively determine whether these receptors serve as the primary functional mediators of propionate’s therapeutic effects in osteoarthritis.

To clarify further the mechanisms by which propionate acts on chondrocytes, we examined its role not only in regulating inflammation but also in autophagy and inflammatory cell death. Autophagy is critical for chondrocyte survival and preservation of ECM integrity. In OA cartilage, reduced autophagic activity contributes to enhanced chondrocyte apoptosis and catabolism (13). Our findings showed that propionate restored autophagy markers in chondrocytes. Notably, pharmacological inhibition of autophagy using 3-MA attenuated the propionate-induced restoration of autophagic activity, suggesting that propionate-mediated reactivation of autophagy may contribute to suppression of inflammatory signaling and prevention of matrix degradation. Importantly, the reduction of phosphorylated RIP1 and RIP3 at the protein level suggests that propionate interferes with activation of the necroptotic signaling cascade rather than simply modulating transcriptional expression of inflammatory mediators. As necroptosis has increasingly been implicated in inflammatory cartilage degeneration (39), these findings indicate that propionate may support chondrocyte survival by promoting autophagy while concurrently limiting inflammatory cell death signaling. Together, this coordinated regulation points toward a broader immunometabolic mechanism through which propionate may influence OA progression. Importantly, functional inhibition of autophagy using 3-MA attenuated the propionate-induced restoration of autophagy markers, providing mechanistic evidence that autophagy activation is not merely associated with, but contributes to, the anti-inflammatory and anti-catabolic effects observed in OA chondrocytes. These findings suggest that autophagy may act as a key upstream regulatory mechanism through which propionate coordinates suppression of inflammatory signaling and preservation of cellular homeostasis.

Although this study demonstrated the disease-modifying effects of propionate in both an OA animal model and human chondrocytes, further investigation is required to clarify the specific signaling pathways involved, particularly whether these effects are mediated through GPR41/43 or other SCFA receptors in chondrocytes and neurons.

In summary, our findings indicate that propionate, a gut microbiota–derived SCFA, alleviates OA-related pain by modulating neurological pathways and mitigates cartilage damage by enhancing chondrocyte autophagy and suppressing inflammatory responses. These results support the concept that targeting the gut microbiota and its metabolites may represent a promising disease-modifying strategy for OA.

## Data Availability

The data presented in the study are deposited in the NCBI Sequence Read Archive (SRA) repository, accession numbers PRJNA1334020 and PRNJA1336958.
