# Supplementary material for: Propionate attenuates osteoarthritis progression by regulating the gut-joint axis
Source: Front Immunol. 2026 Mar 11;17:1717556. doi: 10.3389/fimmu.2026.1717556 (PMC13012969; doi:10.3389/fimmu.2026.1717556)
Supplement: Supplementary file 1 [file DataSheet1.docx]

Supplementary Material

# Supplementary Figure

**
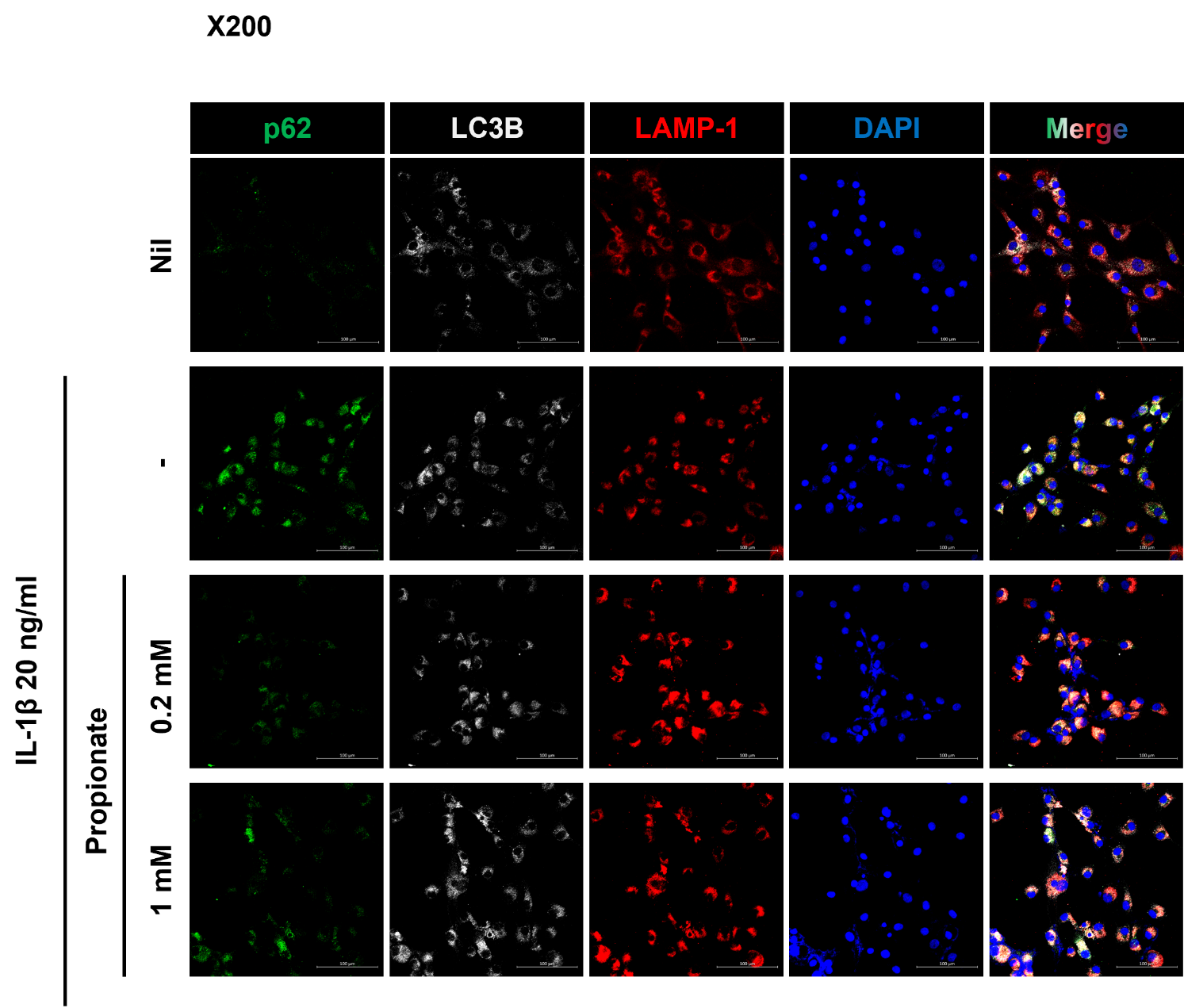
Supplementary Figure S1.** Confocal microscopy of autophagy-related proteins p62 (green), LC3B (white), LAMP-1 (red), and DAPI (blue) in human OA chondrocytes treated with 20 ng/mL IL-1β (magnification ×200).

**
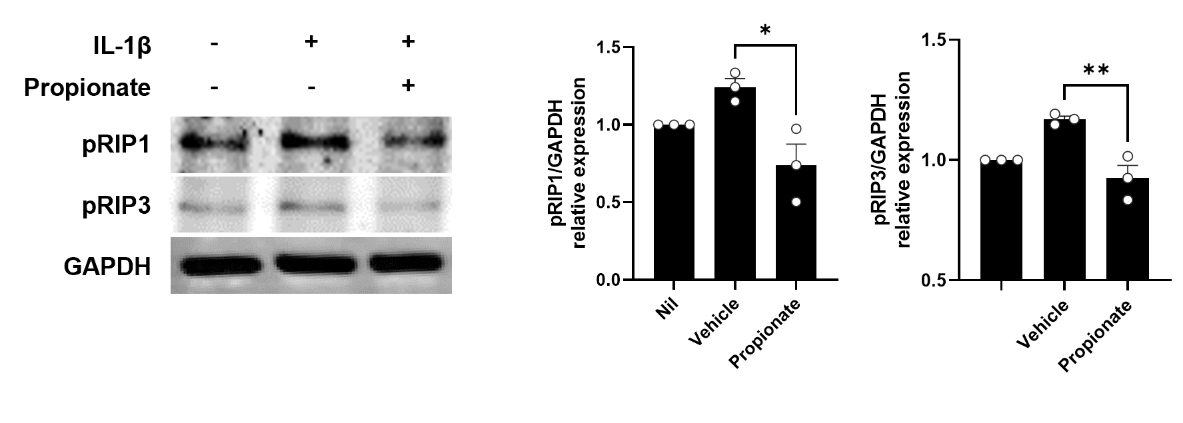
**

**Supplementary Figure S2.** OA chondrocytes were treated with IL-1β (20 ng/mL) in the presence or absence of propionate (0.2 mM). Expression of phospho-RIP1, phospho-RIP3 in OA chondrocytes was analyzed by western blotting (left). GAPDH was used as a loading control. Quantification of pRIP1/GAPDH and pRIP3/GAPDH ratio is shown (right). Statistical significance was determined by Kruskal–Wallis. *p < 0.05.

# Supplementary Table

Supplementary Table S1 Primary antibodies used for immunohistochemistry in rat tissue.

| No. | Tissue | Target | Dilution | Cat.No. | Company |
| --- | --- | --- | --- | --- | --- |
| 1 | DRG | TRPV1 | 1:200 | NB100-1617 | Novus |
| 2 |  | CGRP | 1:400 | ab81887 | Abcam |
| 3 | Joint | IL-1β | 1:400 | NB600-633 | Novus |
| 4 |  | IL-17 | 1:400 | ab79056 | Abcam |
| 5 |  | MCP-1 | 1:600 | ab7202 | Abcam |
| 6 |  | LC3B | 1:400 | ab48394 | Abcam |
| 7 |  | p-mTOR | 1:200 | 2976S | Cell signaling |
| 8 |  | mTOR | 1:100 | 2983S | Cell signaling |
| 9 |  | MMP1 | 1:200 | 10371-2-AP | Proteintech |
| 10 |  | MMP3 | 1:100 | ab52915 | Abcam |
| 11 |  | MMP13 | 1:200 | ab39012 | Abcam |
| 12 |  | iNOS | 1:100 | ab15323 | Abcam |
| 13 |  | RIP3 | 1:200 | PA5-19956 | Introgen |
| 14 |  | p-MLKL | 1:250 | ab19436 | Abcam |
| 15 |  | CASP1 | 1:200 | NB100-56565 | Novus |

Table S2 Primary antibodies used for immunofluorescence in rat tissue and human OA chondrocytes.

| No. | Tissue | Target | Dilution | Cat.No. | Company |
| --- | --- | --- | --- | --- | --- |
| 1 | Intestine | ZO-1 | 1:400 | 40-2200 | Invitrogen |
| 2 |  | OCLN | 1:400 | 331588 | Invitrogen |
| 3 | Chondrocyte | p62 | 1:200 | ab56416 | Abcam |
| 4 |  | LC3B | 1:400 | ab48394 | Abcam |
| 5 |  | LAMP-1 | 1:200 | sc-20011 | Santa Cruz |

Table S3. Primer sequences used for quantitative real-time PCR analysis in human OA chondrocytes.

| No. | Primer | Sequence (5’ to 3’) |
| --- | --- | --- |
| 1 | (H) MMP-1 -S | CTGAAGGTGATGAAGCAGCC |
|  | (H) MMP-1 -AS | AGTCCAAGAGAATGGCCGAG |
| 2 | (H) MMP-3 -S | CTCACAGACCTGACTCGGTT |
|  | (H) MMP-3 -AS | CACGCCTGAAGGAAGAGATG |
| 3 | (H) MMP-9 -S | TGTACCGCTATGGTTACACTCG |
|  | (H) MMP-9 -AS | GGCAGGGACAGTTGCTTCT |
| 4 | (H) RUNX2 -S | CTGAGATTTGTGGGCCGGA |
|  | (H) RUNX2 -AS | GGGGAGGATTTGTGAAGACGG |
| 5 | (H) INOS (NOS2) - S | CCTGAGCTCTTCGAAATCCCA |
|  | (H) INOS (NOS2) - AS | CCCGAAACCACTCGTATTTGG |
| 6 | (H) RIPK1 -S | GACGAAGCCAACTACCATCTT |
|  | (H) RIPK1 -AS | TCTCCTTTCCTCCTCTCTGTT |
| 7 | (H) MLKL-S | AGGAGGCTAATGGGGAGATAGA |
|  | (H) MLKL-AS | TGGCTTGCTGTTAGAAACCTG |
| 8 | (H) CASP1 (caspase-1) -S | TTTCCGCAAGGTTCGATTTTCA |
|  | (H) CASP1 (caspase-1) -AS | GGCATCTGCGCTCTACCATC |
| 9 | (H) ACTB (β-actin) -S | CATGTACGTTGCTATCCAGGC |
|  | (H) ACTB (β-actin) -AS | CTCCTTAATGTCACGCACGAT |
