# Supplementary figures and images for: Propionate attenuates osteoarthritis progression by regulating the gut-joint axis
Source: Front Immunol. 2026 Mar 11;17:1717556. doi: 10.3389/fimmu.2026.1717556 (PMC13012969; doi:10.3389/fimmu.2026.1717556)

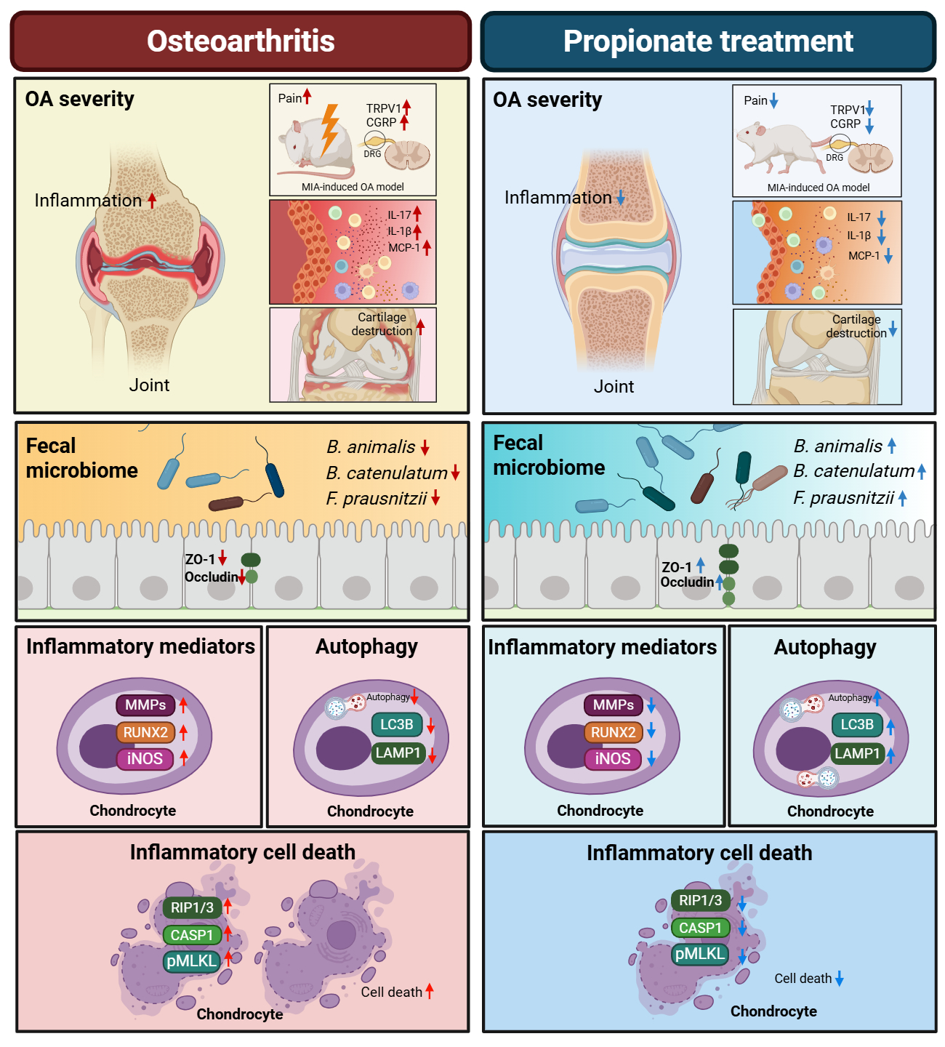

Supplement: Supplementary file 2 [file Image1.tif]
